# Prevalence of vitamin D deficiency and association with metabolic syndrome in a Qatari population

**DOI:** 10.1038/nutd.2017.14

**Published:** 2017-04-10

**Authors:** K Al-Dabhani, K K Tsilidis, N Murphy, H A Ward, P Elliott, E Riboli, M Gunter, I Tzoulaki

**Affiliations:** 1 Department of Biostatistics and Epidemiology, School of Public Health, Imperial College London, London, UK; 2 Department of Hygiene and Epidemiology, University of Ioannina Medical School, Ioannina, Greece; 3 Nutritional Epidemiology Group, International Agency for Research on Cancer; 4 MRC-PHE Centre for Environment and Health, School of Public Health, Imperial College London, London, UK

## Abstract

**Objectives:**

Despite long hours of sunlight in Qatar and other regions of the Middle East, vitamin D deficiency has been rising. In parallel, the prevalence of metabolic syndrome has also been increasing in Qatar. Vitamin D levels have been associated with metabolic syndrome but the data are inconsistent and no studies have addressed these inter-relationships in a Middle Eastern population where the prevalence of these conditions is high. The objective is to investigate the prevalence of vitamin D deficiency and its association with metabolic syndrome and its components in the Qatar Biobank population.

**Methods:**

A cross-sectional study of 1205 participants (702 women and 503 men) from the Qatar Biobank, comprising Qataris and non-Qataris between the ages of 18 and 80 years, was used to perform multivariate linear regression analyses to examine the association between metabolic syndrome and prevalence of vitamin D deficiency (defined as <20 ng ml^−1^ serum vitamin D levels) adjusting for age, sex, ethnicity, season of blood collection, physical activity and education. Odds ratios and 95% confidence intervals were calculated for all analyses.

**Results:**

Approximately 64% of participants were vitamin D deficient (<20 ng ml^−1^) with more men being deficient (68.6%) than women (61.3%). Serum vitamin D was 8% lower in individuals with metabolic syndrome (RR: 0.92, 95%CI: 0.87–0.98, *P*-value: 0.01) compared to individuals without metabolic syndrome. Waist circumference and HDL as well as high triglyceride levels were also significantly positively associated with vitamin D deficiency. No association was found between the other components of metabolic syndrome or diabetes and the presence of vitamin D deficiency.

**Conclusions:**

Vitamin D deficiency is prevalent in this Qatari population. Presence of metabolic syndrome was associated with presence of vitamin D deficiency. Future prospective studies need to be conducted to investigate the potential for causality.

## Introduction

Vitamin D deficiency is highly prevalent worldwide and is associated with many adverse health outcomes.^1, 2^ Vitamin D is acquired in three ways; from sun exposure, diet and supplements; however, the greatest proportion is obtained from sun exposure. For example, exposure to 0.5 minimal erythemal dose is equivalent to supplementing ~3000 IU of vitamin D_3_.^1^ One of the main physiological functions of vitamin D is to maintain calcium and phosphorous levels in the body to sustain various metabolic functions, including bone metabolism.^3^ The most abundant circulating biomarker of vitamin D status is 25-hydroxyvitamin D (25(OH)D), which also has a longer half-life (25 days) compared to the active metabolite; 1,25-dihydroxyvitamin D (7 h).^4^ However, the threshold used to define vitamin D deficiency often varies according to the population and outcome of interest.^5, 6, 7^ The most common definition for optimal 25(OH)D levels is the concentration at which it supresses the parathyroid hormone to its minimum, however, using this definition results in a wide range of minimal optimal values from 12 ng ml^−1^ to 40 ng ml^−1^.^7^

A number of studies have shown that vitamin D levels are inversely associated with the risk of a diverse set of diseases, including several cancers, diabetes and cardiovascular diseases.^8, 9, 10, 11, 12^ The association between vitamin D levels and a range of disease outcomes could be explained by intermediate disease risk factors such as the metabolic syndrome (MetS); a constellation of risk factors, including increased obesity, hypertriglyceridemia, hypertension and diabetes. Some studies have suggested an inverse associations between serum vitamin D and MetS,^2, 12, 13, 14, 15, 16^ while others have not confirmed this observation.^17, 18^ It is also unclear which components of the MetS might drive this association with vitamin D with some studies, suggesting obesity and others glucose haemostasis.^12, 16, 19, 20^

Despite the long hours of sunlight in Qatar and surrounding regions, vitamin D deficiency has been shown to be highly prevalent in this region.^21, 22, 23, 24, 25, 26^ For example, a Kuwaiti and an Emirati study reported that ~98 and 83% (respectively) of the participants had serum 25(OH)D less than 20 ng ml^−1^.^24, 25^ Studies thus far conducted in Qatar on vitamin D were specific to a certain population (elderly and health professionals). At the same time, the prevalence of MetS was measured to be ~26.5%, according to the International Diabetes Federation (IDF) criteria, amongst Qataris aged 20 and over.^27^ Similarly, there was a high prevalence of MetS, according to both the IDF and Adult Treatment Panel (ATPIII) criteria, and its components in neighbouring regions.^28, 29, 30, 31^ Given the high prevalence of these two conditions in the region and the limited epidemiological evidence in this population so far, we aimed to investigate the prevalence of vitamin D deficiency, as well as the association between MetS and its components with vitamin D, in the well-characterised Qatar Biobank.

## Materials and Methods

### Data source

The data for this study was obtained from the Qatar Biobank (QBB), which consisted of 1205 participants; with 702 females and 503 males, at the time of investigation. Participants who were either Qatari or non-Qataris but who were long-term residents of Qatar (that have lived there for over 15 years) were included in the cohort. Recruitment for these participants occurred mostly by personal recommendations of friends and family between December 2012 and February 2014. Participation was voluntary and registration was conducted via online and telephone bookings. A maximum of 10 participants were seen per day. All participants gave informed consent. Detailed methods for recruitment and study design have been reported elsewhere.^32^ Questionnaires on health, lifestyle, and diet were completed. Anthropometric measurements and body composition were also obtained. Biological samples, including blood, a urine sample and a saliva sample, were collected from each participant after the questionnaires were filled out.

A total of 66 clinical biomarkers (including bone and joint markers, coagulation tests, diabetes related tests, differential white cell count, full blood count, sex steroid hormones, lipid profile, minerals and vitamins) were routinely measured for all participants. Haematology and blood biochemistry were analysed by the laboratories at Hamad Medical Corporation (HMC).

### Exposure and outcome assessments

#### Vitamin D assessment

25(OH)D was analysed in one lab at HMC, which is College of American Pathologist (CAP) accredited, using a LIAISON 25 OH Vitamin D TOTAL Assay, where serum 25(OH)D_2_ and 25(OH)D_3_ were measured. In this study, vitamin D deficiency was defined by the US Endocrine Society (USES) guidelines as <20 ng ml^−1^ (50 nmol l^−1^) in circulation, while individuals with 21–29 ng ml^−1^ were considered insufficient, and individuals with >30 ng ml^−1^ were considered sufficient.^33^ To convert between ng ml^−1^ and nmol l^-1^ of 25(OH)D, multiply the concentration of 25(OH)D in ng ml^−1^ by 2.5.

#### Anthropometric measures

Weight, height, body mass index (BMI), and waist and hip circumference were all measured by a trained nurse. Participants wore gowns or light weight clothing, and shoes and socks were removed. Weight was measured using the TANITA BC-418 MA instrument; a multi-frequency segmental body composition analyser, or a digital floor scale Seca-876 for manual weight measurement when TANITA was contraindicated. BMI was calculated as weight in kilogram divided by height in metres squared (kg m^−2^). Waist and hip measurements were measured using a non-stretchable sprung measuring tape by Seca. The waist was identified as the smallest part of the trunk after folding their arms across their chest. If it was not possible to find a natural indent of the trunk, the circumference around the umbilicus was measured in centimetres. For the hip measurement, the Seca measuring tape was placed at the widest part of the hips and measured in centimetres. Waist-to-hip ratio was calculated as waist in centimetres divided by hip circumference also in centimetres.

#### Metabolic syndrome components

Metabolic syndrome was defined according to the new IDF definition as being centrally obese (defined as waist circumference ⩾94 cm for males and ⩾80 cm for females) as well as at least two of the following four factors: (1) raised triglycerides (⩾1.7 mmol l^−1^),^2^ (2) reduced HDL cholesterol (<1.03 mmol l^−1^ in males and <1.29 mmol l^−1^ for females), (3) raised blood pressure (BP) (systolic BP ⩾130 mm Hg or diastolic BP ⩾85 mm Hg), (4) raised fasting plasma glucose (defined as HbA1c levels⩾5.7 mmol l^−1^).^34^ HbA1c was measured with turbidimetric inhibition immunoassay (TINIA). Cholesterol was measured using CHOD-PAP GEN2 STAND ID/MS, HDL was measured using the HDL–C plus third generation, LDL was measured using the LDL-C plus second generation and triglyceride was measured using GPO-PAP.

Blood pressure was measured by a trained nurse using an Omron M10-IT automated upper arm blood pressure monitor with arm cuffs of different sizes. The reading was collected twice if the results were similar, but if different (differed by more than 5 mm Hg) a third measurement was performed. The participant rested for 30 s between the measurements. If the measurements did not differ, the average of the first two measurements was taken. However, if the measurements did differ, the average of the second and third measurement was used. Diabetes was reported in two ways: (1) self-reported; where 15.4% reported having diabetes and (2) laboratory measurements of HbA1c levels (⩾6.5% was considered evidence for diabetes),^35^ where 17.4% of participants were considered diabetic.

### Statistical analysis

To test the difference in the distributions of categorical variables, a *χ*^2^ test was performed, while a *t*-test was performed to test the differences between a categorical and a continuous variable after stratifying by sex. A geometric mean for serum 25(OH)D was calculated by performing a one-way Analysis of Covariance (ANCOVA) adjusting for sex, age and season of blood collection. Partial Spearman's correlation coefficient was used to measure the cross-sectional association between anthropometric measurements, circulating lipids, haemoglobin A1c (HbA1c), glucose, insulin, C-peptide, folate, vitamin D, calcium and SHBG.

Vitamin D was modelled as a continuous measurements. Metabolic syndrome and its components were categorised into having abnormal levels or normal levels based on the IDF definition.^34^ Univariate (crude) and multivariable linear regression models were used to calculate the regression coefficients and associated 95% confidence intervals (CI). Multivariable model (Model 2) was adjusted for age (continuous), sex, ethnicity, education, physical activity and season. Participants with missing ethnicity values (*n*=242) were excluded from all analyses.

## Results

### Descriptive characteristics

Approximately 58% of the study population were females; mean age was similar between males and females (Table 1). There were several differences between males and females with regards to anthropometric factors: men had more visceral fat (97 cm) than women (85 cm) and also reported to smoke more and exercise more frequently (39% and 19 h per week, respectively) than women (12% and 11 h per week, respectively). BMI as well as the prevalence of diabetes (~15%) and metabolic syndrome (~28%) were similar between males and females (Table 1).

Approximately 64% of the participants in this study were vitamin D deficient (<20 ng ml^−1^) with slightly more men (69%) being vitamin D deficient compared to women (61%). Another 25% of the population had insufficient vitamin D levels. Table 1 reports the prevalence of stages of vitamin D deficiency according to USES definitions.^33^ The percentage of people reported taking vitamin D supplements was high with 49% of females and 25% of males (Table 1). Women were more likely to undergo vitamin D screening, and take vitamin D and calcium supplements compared to men.

Table 2 shows crude and adjusted mean vitamin D levels across categories of participant characteristics. Females reported a higher concentration of serum 25(OH)D compared to males in both the adjusted and unadjusted mean 25(OH)D, although this difference was not statistically significant. Also, higher 25(OH)D levels were observed in older compared to younger participants. The remaining characteristics did not show differences according to 25(OH)D levels.

### Associations between metabolic syndrome, vitamin D deficiency and serum 25(OH)D levels

Table 3 reports the association between metabolic syndrome with the risk of vitamin D deficiency (<20 ng ml^−1^) and serum 25(OH)D levels. There was a significant positive association between the presence of MetS and vitamin D deficiency after adjusting for Model 2 (OR: 1.54, 95%CI: 1.09–2.18) (Supplementary Table 1). However, age was the main confounder responsible for the statistical significant association between MetS and its components with 25(OH)D concentration. The presence of MetS was associated with lower 25(OH)D levels (RR per ng ml^−1^: 0.92, 95%CI: 0.86–0.98). Components of the MetS that were inversely associated with serum 25(OH)D levels, including high waist circumference (high vs normal, RR per ng ml^−1^: 0.94, 95%CI: 0.88–0.99) and elevated triglycerides (high vs normal, RR: 0.90, 95%CI: 0.84–0.95). While increasing levels of HDL was positively associated with 25(OH)D levels (per mmol l^−1^ increase RR: 1.12, 95%CI: 1.03–1.21). Elevated blood pressure showed weak inverse association with vitamin D; the association was stronger with the presence of vitamin D deficiency (OR for high vs normal: 1.60, 95%CI: 1.06–2.42, *P*-value: 0.03) (Supplementary Table 1) compared to 25(OH)D levels; (RR per one unit increase: 0.93, 95%CI: 0.86–1.00, *P*-value: 0.06). The association between vitamin D deficiency or 25(OH)D levels with presence of diabetes was not statistically significant.

## Discussion

In this unique study in Qatar, we showed that despite being a country with high levels of sun exposure, the prevalence of vitamin D deficiency is very high (64%),with the majority of the examined population showing signs of deficiency. This is consistent with previous observations which estimated the prevalence of vitamin deficiency (<20 ng/ml) to range between 72–87%.^22, 26^ At the same time, the percentage of participants reporting supplementation for vitamin D, especially in women was very high (49%), higher than that observed in white populations,^36, 37, 38^ potentially showing a greater awareness of vitamin D deficiency in this population.

Approximately 64% of the participants in the Qatar Biobank were vitamin D deficient (<20 ng ml^−1^), with slightly more men being deficient compared to women. Previous evidence from studies in the Middle East region supports higher level of vitamin D in males compared to females. A Saudi Arabian study performed on 10 709 participants reported more females being severely vitamin D deficient (<10 ng ml^−1^) compared to males.^23^ A Bahraini and Lebanese study also reported lower mean serum vitamin D in females compared to males.^21, 39^ One hypothesis for this difference could be explained by vitamin D supplementation, as women in the Qatar Biobank were more likely to take vitamin D supplements compared to men. This was not reported in both the Saudi and Bahraini studies due to exclusion of participants that consumed vitamin D supplements.^21, 23^

MetS was significantly associated with the risk of vitamin D deficiency in the Qatar BioBank. Several cross-sectional and few prospective studies have reported similar positive associations between vitamin D deficiency and the risk of metabolic syndrome.^12, 15, 40, 41, 42^ In addition, prospective studies from the PROMISE cohort and the Australian Diabetes, Obesity and Lifestyle (Aus-Diab) study, reported significant inverse associations between continuous serum vitamin D and overall MetS, based on the IDF criteria.^12, 40^ Studies examining the direction of the effect between vitamin D, cholesterol and lipids suggest that lipids levels and BMI may be a cause for decreased vitamin D and not vice versa.^43, 44^ Other cross-sectional studies did not support these associations.^45, 46^ However, this might have been due to the small sample sizes^45, 47^ and small variation in the exposure (e.g. high levels of vitamin D in the study by Reis *et al.* in both men and women).^46^ Studies on Middle Eastern population where the metabolism of vitamin D could be different due to different lifestyles and darker skin pigmentation, are very limited and our results support an inverse association between the two examined phenotypes.

Many mechanisms have been proposed to explain the association between vitamin D and future risk of MetS component. Since vitamin D is fat soluble and could be stored in adipose tissue, it can be sequestered in the subcutaneous fat in obese individuals, reducing the levels of circulating vitamin D in the blood leading to less release of vitamin D into the blood.^48, 49^ Vitamin D has also been shown to inhibit the release of cytokines from the immune cell, which is harmful to β cells.^50^

Obesity, measured as BMI, waist circumference, and waist-to-hip ratio were positively associated with vitamin D deficiency with stronger associations observed with waist circumference. Similar results were found in both prospective and cross-sectional studies, including the Aus-Diab study,^40^ the British Birth Cohort,^47^ Middle Eastern populations,^25, 51^ and in other populations around the world.^15, 52, 53^ Conversely, several cross-sectional studies did not report a correlation between obesity and vitamin D deficiency.^45, 46, 54^ However, these are mainly small studies with limited power to observe associations.^45, 46^ We also showed that high triglyceride levels were associated with lower levels of vitamin D. Studies also report significant inverse associations between vitamin D with high triglyceride levels.^52, 55^ However, when stratified by sex, only the association in men remained significant in the Ling *et al.* study.^52^ On the contrary, Reis *et al.* reported a positive trend between vitamin D deficiency and high triglyceride levels only in women.^46^

This is the first study of vitamin D and metabolic syndrome performed in the Qatari population and long-term residents (⩾15 years living in Qatar) with high-quality data, which involves collection of extensive questionnaire information, clinical phenotyping and biological samples.^32^ Qatar Biobank, as of yet is not considered to have a fully representative sample, since the most common source of participant recruitment is through word of mouth to friends and families.^32^ However, the population of our study shows small differences with the Qatar Stepwise Survey (QSS), which is based on the WHO STEPS survey and consists of 2 496 participants. In the QSS, measurements of height, weight, and BMI were similar to this study. However, in Qatar Biobank, over 60% of the participants had a university degree compared to 35% of the QSS,^56^ suggesting a higher representation of more educated and higher socioeconomic status individuals in Qatar Biobank.

Since this study is cross-sectional, the directionality of the relationship between adiposity, vitamin D, metabolic syndrome, and diabetes could not be elucidated. The non-significant results for the association between diabetes and vitamin D deficiency, may have been due to the relatively small sample size and low response rate for some questions regarding physical activity, smoking, supplements and so on. It could also have been due to other confounding factors such as specific medication use and dosage of supplements, which were not captured. Approximately 84% of participants were missing data on medication use for hypertension and 68% of participants were missing for medication on cholesterol usage, which may have caused some misclassification. Additionally, the low levels of circulating vitamin D found in this population may have been insufficient to observe any inverse relationships with metabolic syndrome components and diabetes.

To conclude, the findings from this study support a positive association between the presence of metabolic syndrome and vitamin D deficiency. We also observed that MetS components, such as obesity and high triglyceride, were inversely associated with circulating serum vitamin D levels. Future prospective studies should elucidate the potential causal association between vitamin D and MetS using Mendelian randomisation approaches or through supplementation with Vitamin D. Moreover, mechanistic studies should concentrate on identifying pathophysiological pathways and molecular mechanisms linking vitamin D deficiency and metabolic syndrome.

## Figures and Tables

**Table 1:** Main characteristics of Qatar Biobank study participants stratified by sex

*Characteristic*	*Females (*N=*702)*	*Males (N*=*503)*	*Total (N*=*1205)*
	*Mean (s.d.)*	*Mean (s.d.)*	*Mean (s.d.)*
Age (IQR) (*N*=1205)	39.3 (28–50)	40.8 (30–51)	39.9 (29–50)
Sex^a^ (%) (*N*=1205)	58.2	41.8	
BMI (kg m^−2^) (*N*=1199)	29.0 (6.8)	28.7 (5.5)	28.9 (6.3)
Height (cm) (*N*=1203)	158.4 (6.0)	172.5 (6.6)	164.3 (9.3)
Weight (kg) (*N*=1199)	72.6 (17.2)	85.5 (18.1)	78.0 (18.7)
Waist circumference (cm) (*N*=1994)	85.0 (14.7)	97.2 (13.6)	90.1 (15.5)
WHR (*N*=1194)	0.8 (0.1)	0.9 (0.1)	
			
*Education*^a^ *(%) (*N=*1202)*			
Less than primary school	4.0	0.2	2.4
Primary school	4.3	2.0	3.3
Secondary school	5.3	7.2	6.1
Technical/professional school	22.1	22.9	22.5
University/postgraduate	74.2	67.7	65.7
			
*Smoking status*^a^ *(%) (*N=*668)*			
Never smoked	86.0	47.7	59.6
Stopped smoking	1.4	13.7	9.9
Once/twice	7.2	6.5	6.7
Occasionally	1.9	10.0	7.5
Smoke on most/all days	3.4	22.1	16.3
			
*Ethnicity*^a^ *(%) (*N=*954)*			
Non-Qatari	18.6	39	27.4
Qatari	81.4	61.0	72.6
			
*Season of blood draw*^a^ *(%) (*N=*1205)*			
Winter^b^	33.0	33.8	33.4
Spring^b^	22.9	33.0	27.1
Summer^b^	14.2	13.3	13.9
Fall^b^	29.8	19.9	25.6
MET score (h per week) (*N*=1205)	10.8 (24.8)	19.5 (43.8)	14.5 (34.3)
Self-reported diabetic^a^ (%) (*N*=1198)	14.6	16.4	15.3
Metabolic syndrome^a^ (%) (*N*=1205)	29.2	27.8	28.6
Vitamin D supplementation^a^ (%) (*N*=1205)	49.0	24.8	38.9
			
*25-hydroxyvitamin D ng ml^−1^*^a^ *(%) (N=1176)*			
Severely deficient (<10 ng ml^−1^)	10.6	5.8	8.6
Deficient (10-<20 ng ml^−1^)	50.7	62.8	55.8
Insufficient (20–<30 ng ml^−1^)	26.1	24.7	25.5
Sufficient (⩾30 ng ml^−1^)	12.7	6.6	10.1

Abbreviations: MET, Metabolic equivalent; WHR, waist-to-hip ratio.

a Categorical variables with (%) indicate percentages rather than means and standard deviation. The percentages were taken after removing missing, prefer not to answer, and I do not know categories.

b Seasons defined as winter: December-February, spring: March-May, summer: June-August, and fall: September–November.

**Table 2:** Unadjusted and adjusted mean 25-hydroxyvitamin D levels by participant characteristics

*Characteristics*	*Unadjusted mean 25(OH)D (s.d.)*^a^	P*-value*	*Adjusted mean 25(OH)D (s.d.)*^b^	P*-value*	*P-Trend*
Sex (*N*=1176)		0.06			0.08
Female (*N*=679)	19.0 (10.0)		17.0 (0.1)		
Male (*N*=497)	18.0 (9.0)		16.5 (0.1)	0.08	
Age (*N*=1176)		<0.001			<0.001
<25 (*N*=140)	15.1 (9.1)		13.5 (0.0)		
25–34 (*N*=352)	16.7 (9.2)		15.0 (0.0)	0.01	
35–44 (*N*=224)	18.4 (9.5)		16.6 (0.0)	<0.001	
45–54 (*N*=283)	20.3 (10.0)		18.7 (0.0)	<0.001	
⩾55 (*N*=177)	22.5 (8.3)		21.1 (0.0)	<0.001	
Education (*N*=1173)		<0.001			0.46
Less than primary school (*N*=29)	23.1 (8.7)		21.4 (0.1)		
Primary school (*N*=39)	23.9 (10.7)		22.1 (0.1)	0.53	
Secondary School (*N*=69)	19.3 (6.8)		18.1 (0.1)	0.72	
Technical/Professional school (*N*=263)	17.5 (11.4)		15.5 (0.1)	0.17	
University/Postgraduate (*N*=773)	18.3 (9.0)		16.8 (0.1)	0.64	
Smoking status (*N*=656)		0.14			0.92
Never smoked (*N*=388)	18.2 (10.4)		16.4 (0.1)		
Stopped smoking (*N*=66)	19.5 (10.1)		17.8 (0.1)	0.21	
Once/twice (*N*=45)	17.7 (9.6)		15.7 (0.1)	0.99	
Occasionally (*N*=50)	19.2 (9.5)		17.3 (0.1)	0.30	
Smoke on most/all days (*N*=107)	16.7 (6.7)		15.6 (0.1)	0.86	
Ethnicity (*N*=934)		<0.001			0.29
Non-Qatari (*N*=261)	17.6 (6.5)		17.2 (0.1)		
Qatari (*N*=673)	18.5 (9.2)		16.7 (0.1)	0.29	
Season blood draw (*N*=1176)		0.99			0.43
Winter^c^ (*N*=400)	18.5 (10.2)		17.1 (0.1)		
Spring^c^ (*N*=318)	18.6 (8.8)		16.8 (0.1)	0.46	
Summer^c^ (*N*=164)	18.2 (9.1)		16.8 (0.1)	0.62	
Autumn^c^ (*N*=294)	18.9 (9.7)		16.4 (0.1)	0.41	
MET score (h per week) (*N*=1176)		0.24			0.15
0 (*N*=415)	18.8 (10.3)		16.8 (0.1)		
>0–9.99 (*N*=401)	18.5 (8.2)		16.9 (0.1)	0.77	
10–49.99 (*N*=281)	18.0 (7.5)		16.5 (0.1)	0.73	
⩾50 (*N*=79)	20.4 (14.6)		17.9 (0.1)	0.02	
Vitamin D supplementation		<0.001			<0.001
None	15.4 (6.4)		14.3 (0.1)		
Yes	23.6 (11.4)		21.7 (0.1)		

Abbreviation: MET, metabolic equivalent.

a An ANCOVA test was used to measure significance.

b Mean vitamin D was adjusted for age, sex and season of blood collection.

c Seasons defined as Winter: December–February, Spring: March–May, Summer: June–August, and Autumn: September–November.

**Table 3:** Linear regression analyses between metabolic syndrome and its components with vitamin D

	*Per* ng/ml *increase of vitamin D*			
	*Model 1: Unadjusted*		*Model 2*^a^	
	*β (95%CI)*	P*-value*	*β (95%CI)*	P*-value*
*MetS*^b^				
Normal	Ref			
MetS	1.09 (1.03–1.15)	0.01	0.92 (0.87–0.98)	0.01
				
*WC*^b^				
Normal	Ref			
High	1.05 (1.00–1.11)	0.04	0.94 (0.88–0.99)	0.03
Per 10cm increase	1.00 (0.99–1.02)	0.53	0.96 (0.94–0.98)	0.001
				
*TG*^b^				
Normal	Ref			
High	0.98 (0.92–1.04)	0.52	0.90 (0.84–0.95)	0.001
Per 10% increase	1.00 (1.00–1.01)	0.18	0.99 (0.99–1.00)	0.002
				
*BP*^b^				
Normal	Ref			
High	1.05 (0.97–1.12)	0.21	0.93 (0.86–1.00)	0.06
Per 10 mm Hg SBP	1.04 (1.02–1.05)	<0.001	0.99 (0.97–1.01)	0.38
Per 10 mm Hg DBP	1.04 (1.01–1.06)	0.003	0.96 (0.94–0.99)	0.02
				
*HDL*^b^				
Normal	Ref			
Low	1.08 (1.03–1.14)	<0.001	0.99 (0.94–1.05)	0.81
Per mmol/l increase	1.06 (0.99–1.13)	0.10	1.12 (1.03–1.21)	0.01
				
*Diabetes- self reported*				
No	Ref			
Yes	1.15 (1.07–1.23)	<0.01	1.00 (0.92–1.08)	0.95
				
*Diabetes–measured*^c^				
No	Ref			
Yes	1.13 (1.05–1.20)	<0.01	0.99 (0.92–1.06)	0.76

Abbreviations: BMI, body mass index; BP, blood pressure;

HDL, high-density lipids; MetS, metabolic syndrome, according to the IDF criteria; TG, triglyceride; WC, waist circumference; WHR, waist-to-hip ratio.

Per 1 ng ml^−1^ increase in serum vitamin D levels. Vitamin D levels were logarithmic transformed in the model and the β were then exponentiated

a Model 2: Linear regression adjusted for age, sex, ethnicity, MET score, education and season of blood draw.

b Cutoffs were defined according to the International Diabetes Federation.

c Cutoff was defined according to the American diabetes association.
