# Supplementary material for: Prevalence of vitamin D deficiency and association with metabolic syndrome in a Qatari population
Source: Nutr Diabetes. 2017 Apr 10;7(4):e263–. doi: 10.1038/nutd.2017.14 (PMC5436094; doi:10.1038/nutd.2017.14)
Supplement: Supplementary Information [file nutd201714x1.docx]

**Supplemental Tables**

Supplemental Table 1: Logistic regression analyses between Metabolic Syndrome and its components with vitamin D deficiency

|  | Vitamin D deficiency (<20ng/mL) | | | |
| --- | --- | --- | --- | --- |
|  | Model 1: Unadjusted |  | Model 2* | |
|  | OR (95%CI) | P-value | OR (95%CI) | P-value |
| **MetS‡** |  |  |  |  |
| Normal | Ref |  | Ref |  |
| MetS | 0.67 (0.52 - 0.87) | <0.01 | 1.54 (1.09 - 2.18) | 0.01 |
| **WC‡** |  |  |  |  |
| Normal | Ref |  | Ref |  |
| High | 0.77 (0.60 - 0.98) | 0.03 | 1.39 (1.01 - 1.92) | 0.04 |
| **TG‡** |  |  |  |  |
| Normal | Ref |  | Ref |  |
| High | 1.22 (0.91 - 1.62) | 0.18 | 2.30 (1.58 - 3.34) | <0.01 |
| **BP‡** |  |  |  |  |
| Normal | Ref |  | Ref |  |
| High | 0.98 (0.70 - 1.37) | 0.92 | 1.60 (1.06 - 2.42) | 0.03 |
| **HDL‡** |  |  |  |  |
| Normal | Ref |  | Ref |  |
| Low | 0.70 (0.55 - 0.89) | <0.01 | 1.01 (0.75 - 1.37) | 0.93 |
| **Diabetes- self reported** |  |  |  |  |
| No | Ref |  | Ref |  |
| Yes | 0.58 (0.42 - 0.80) | <0.01 | 1.23 (0.81 - 1.86) | 0.33 |
| **Diabetes –measured** Ϫ |  |  |  |  |
| No | Ref |  | Ref |  |
| Yes | 0.61 (0.45 - 0.83) | <0.01 | 1.24 (0.83 - 1.84) | 0.30 |

*Model 2: Logistic regression adjusted for age, sex, ethnicity, MET score, education, and season of blood draw

‡WC: waist circumference, WHR: waist-to-hip ratio, BMI: body mass index, MetS: Metabolic Syndrome, according to the IDF criteria, TG: triglyceride, BP: blood pressure, HDL: high density lipids

**‡**Cutoffs were defined according to the International Diabetes Federation

Ϫ Cutoff was defined according to the American diabetes association
